# Novel Betaherpesviruses in Neotropical Bats on the Caribbean Island of St. Kitts: First Report from Antillean Tree Bats (*Ardops nichollsi*) and Evidence for Cross-Species Transmission

**DOI:** 10.3390/microorganisms12122603

**Published:** 2024-12-16

**Authors:** Jessica L. Kulberg, Sarah Hooper, Yashpal S. Malik, Souvik Ghosh

**Affiliations:** 1 Department of Biomedical Sciences, Ross University School of Veterinary Medicine, Basseterre P.O. Box 334, Saint Kitts and Nevis; jessicakulberg@students.rossu.edu (J.L.K.); SHooper@rossvet.edu.kn (S.H.); 2 ICAR-Indian Veterinary Research Institute, Mukteswar 263168, Uttarakhand, India; malikyps@gmail.com

**Keywords:** *Ardops nichollsi*, bats, Caribbean Island, herpesvirus DNA polymerase, interspecies transmission, *Molossus molossus*, putative novel betaherpesviruses, virus–host coevolution

## Abstract

To date, limited information is available on herpesviruses in bats from the Caribbean region. We report here high detection rates (24.24%, n = 66) of herpesviruses in oral samples from apparently healthy bats (*Ardops nichollsi* (75%, 9/12) and *Molossus molossus* (28%, 7/25)) on the Lesser Antillean Island of St. Kitts. Based on analysis of partial DNA polymerase (DPOL) sequences (~225 amino acid (aa) residues), we identified two distinct groups of herpesviruses (BO-I and -II) that were unique to *A. nichollsi* and *M. molossus*, respectively. Within the subfamily *Betaherpesvirinae*, the BO-I DPOL sequences shared low deduced aa identities (<70%) with other herpesviruses, and phylogenetically, they formed a distinct cluster, representing a putative novel betaherpesvirus. The BO-II DPOL sequences were closely related to a putative novel betaherpesvirus from a *M. molossus* in Lesser Antillean Island of Martinique, indicating possible transmission of herpesviruses by bat movement between the Caribbean Islands. Phylogenetically, the BO-I and -II betaherpesviruses exhibited species-specific (*A. nichollsi* and *M. molossus*, respectively) as well as family-specific (*Phyllostomidae* and *Molossidae*, respectively) clustering patterns, corroborating the hypothesis on host specificity of betaherpesviruses. Interestingly, a single *M. molossus* betaherpesvirus strain clustered with the *A. nichollsi* betaherpesviruses, indicating possible interspecies transmission of herpesviruses between *Phyllostomidae* and *Molossidae*. To our knowledge, this is the first report on detection of herpesviruses from Antillean tree bats (*A. nichollsi*), expanding the host range of betaherpesviruses. Taken together, the present study identified putative novel betaherpesviruses that might be unique to chiropteran species (*A. nichollsi* and *M. molossus)*, indicating virus–host coevolution, and provided evidence for interspecies transmission of betaherpesviruses between chiropteran families.

## 1. Introduction

Members of the family *Orthoherpesviridae* (Order *Herpesvirales*) are double-stranded DNA viruses that have been reported in a wide variety of mammalian and avian species and in reptiles [1]. Orthoherpesviruses have been associated with a broad spectrum of clinical conditions (ranging from asymptomatic infection to peracute disease) in different host species [1,2,3]. Taxonomically, the family *Orthoherpesviridae* is composed of three subfamilies: *Alpha*-, *Beta*- and *Gammaherpesvirinae*, consisting of five, five, and seven genera, respectively [1]. In addition, the NCBI Taxonomy database has listed several herpesvirus sequences as ‘unclassified viruses’ within each of the subfamilies [4]. Orthoherpesviruses generally appear to coevolve with their hosts, although host switching events have been implicated to play important roles in the evolution of some of the viruses [1,5,6,7].

Bats (Order *Chiroptera*, the second largest mammalian order) exhibit significant viral diversity and are well-recognized reservoirs of important zoonotic pathogens [8]. Since the first isolation of cytomegalovirus-like particles from *Myotis lucifugus* (little brown bat) in 1996 [9], herpesviruses/herpesvirus sequences have been reported in at least 109 chiropteran species (representing 45 genera and 10 families) [10]. In most of these studies, herpesviruses were detected in apparently healthy bats [10,11,12,13,14,15,16,17]. To date, herpesvirus species representing *Alpha*-, *Beta*- and *Gammaherpesvirinae*, and several unclassified/novel herpesviruses/herpesvirus sequences (currently distributed among the three orthoherpesvirus subfamilies in the NCBI Taxonomy database) have been identified in bats, revealing the high diversity of herpesviruses circulating in chiropteran populations worldwide [1,4,10,18]. Phylogenetically, many chiropteran herpesviruses have been shown to exhibit host-specific clustering patterns, corroborating the hypothesis on coevolution of herpesvirus with its host species [6,10,12,13,14,15,16,17]. On the other hand, phylogenetic studies have also indicated possible host-switching/cross-species transmission events between different chiropteran families/subfamilies/genera and even between bats and other mammals in the evolution of some of the chiropteran herpesviruses [5,7,10,14,15,17,19,20,21].

Although there is no conclusive evidence on interspecies transmission of herpesviruses from bats to humans and other animals [6,10], experimentally, chiropteran herpesviruses have been shown to replicate in human, monkey, feline, and porcine cell lines [22] and cause lethal infection in mice [23], indicating their potential to infect other mammals [10]. Since >1400 chiropteran species have been identified so far [24], our current knowledge on bat herpesviruses might not reflect the true diversity of these viruses within the order *Chiroptera*, warranting further studies in unexplored bat species and in underrepresented geographical regions, such as the Caribbean Islands.

The Caribbean Islands (Bahamas, the Greater and Lesser Antilles) are habitat to at least 61 chiropteran species [25], including certain species that are unique to the region, such as *Ardops nichollsi* (Antillean tree bat) [25,26]. To date, limited virological studies have been conducted in chiropteran species from the Caribbean region and were primarily focused on zoonotic viruses, whilst information on other important bat-borne viruses, especially those of veterinary significance, is lacking [27]. Although zoonotic viruses, such as alphacoronaviruses, chikungunya virus, Eastern equine encephalitis virus, rabies virus, St. Louis encephalitis virus, Tacaribe virus, and Venezuelan equine encephalitis virus, have been documented in bats from the Caribbean region, these reports were based on preliminary serological evidence or limited molecular epidemiological data [27]. Taken together, these observations highlighted the gaps in virology research in Caribbean bat populations, underscoring the importance of large-scale surveillance studies and detailed molecular characterization of bat-borne viruses of human and/or veterinary relevance in the different Caribbean Islands.

To date, limited information is available on herpesviruses in bats from the Caribbean region. Based on analysis of herpesvirus partial DNA polymerase (DPOL) and glycoprotein B (gB) sequences, a study from Martinique reported novel beta- and gammaherpesviruses in *Molossus molossus* (velvety free-tailed bat) and novel gammaherpesviruses in *Sturnira angeli* [13]. In a recent study from Puerto Rico, 30.4% of oral swabs from 1086 bats (representing eight Chiropteran species) tested positive for herpesviruses [28]. The resultant herpesvirus sequences (~178 bp DPOL sequences) were categorized into 43 operational taxonomic units (OTUs), with 13 herpesvirus OTUs identified in more than one bat species [28]. In the present study, we report putative novel herpesviruses related to betaherpesviruses in bats (*A. nichollsi* and *M. molossus*) from the Caribbean Island of St. Kitts. To our knowledge, this is the first report on detection and molecular characterization of herpesviruses in Antillean tree bats (*A. nichollsi*).

## 2. Materials and Methods

### 2.1. Sampling

The present study was based on oral swabs obtained from 66 apparently healthy bats (representing 4 Chiropteran species: *Artibeus jamaicensis* (n = 17), *A. nichollsi* (n = 12), *Brachyphylla cavernum* (n = 12), and *M. molossus* (n = 25)) on the island of St. Kitts during December 2020–October 2022. All activities related to capture (using mist nets) and sampling of bats were performed under a St. Kitts and Nevis Access Benefit and Sharing (ABS) Permit issued to the Ross University School of Veterinary Medicine (RUSVM), St. Kitts, and an approved IACUC protocol (RUSVM IACUC # 20.09.24). Mist net procedures were carried out following the ‘Range-wide Indiana Bat and Northern Long-eared Bat Survey Guidelines’ outlined by the US Fish and Wildlife Service [29].

Mist nets were placed in forests and urban habitats on different parts of St. Kitts. Prior to deployment of mist nets, forest and urban habitats were scouted by foot or vehicle to identify potential mist netting locations. Good mist netting locations were identified as having one or more of the following characteristics: (i) non-moving or slow-moving water sources with appropriate swooping lengths (such as pools or deep puddles >3 m in length and <18 m in width), (ii) a travel corridor, defined as a corridor clear of any debris that bats can easily fly through and surrounded by rock walls or dense rainforest and overhanging branches, or (iii) roosts in man-made structures that allow net placement immediately outside the roost. Once a potential location was identified, acoustic detectors (SM4, Wildlife Acoustics, Maynard, MA, USA) were placed for 4 to 7 nights to confirm high bat activity prior to mist netting. The captured bats were gently removed from the mist nets and placed into individual sterile cloth bags. A single oral sample was obtained from each bat using a sterile swab (MicroTest™ M4RT, Thermo Fisher Scientific Inc., Waltham, MA, USA) and immediately transferred into a sterile tube containing viral transport medium (MicroTest™ M4RT, Thermo Fisher Scientific Inc., Waltham, MA, USA). The bats were released after sampling. All samples were kept on ice during sampling activities and eventually shifted to a −80 °C freezer at the RUSVM research laboratory. In between sampling sites, all equipment were disinfected by boiling, 70% isopropyl alcohol, and/or 10% bleach (sodium hypochlorite) solution.

All sampled bats were identified morphometrically by an experienced field biologist (S.H.) following established guidelines [30,31]. The species identification of bats that tested positive for herpesviruses were further confirmed by sequencing of the mitochondrial DNA cytochrome *b* (cyt-*b*) gene, as described previously [24,32].

### 2.2. Amplification of Herpesvirus DNA in Bats

Total DNA was extracted from oral samples using the QIAamp DNA Mini Kit (Qiagen Sciences, Germantown, MD, USA) following the manufacturer’s instructions. The samples were screened for presence of herpesviral DNA by a *pan*-herpesvirus DPOL nested PCR assay (based on amplification of a short region (~215–315 bp) of the herpesvirus DPOL gene) that has been successfully employed to detect genetically diverse herpesviruses (alpha-, beta- and gammaherpesviruses) in various animal species, including bats [7,12,13,15,20,33]. Longer DPOL sequences (~680 nucleotide (nt) residues) were obtained from the bat herpesviruses in St. Kitts (henceforth designated as ‘BO herpesviruses’) using a combination of the external forward (primer DFA) and reverse (KG1) primers from the screening PCR assay [33], or forward primer 4679F and primer KG1. The forward primer 4679F (5′-TTYGCBAGYCTGTAYCCGTC-3′, corresponding to nt 4679-nt 4698 of bat herpesvirus BatBHV-2, GenBank accession number AB517983) was designed in this study following multiple alignment of DPOL sequences from chiropteran and other mammalian betaherpesviruses. Sterile water was used as the negative control in all PCR assays.

### 2.3. Nucleotide Sequencing

The Wizard^®^ SV Gel and PCR Clean-Up kit (Promega, Madison, WI, USA) was used for purification of PCR products. Nucleotide sequences were obtained by Sanger chemistry using the ABI Prism Big Dye Terminator Cycle Sequencing Ready Reaction Kit (Applied Biosystems, Foster City, CA, USA) on an ABI 3730XL DNA Analyzer (Applied Biosystems, Foster City, CA, USA). Nucleotide sequences were determined in both directions.

### 2.4. Analyses of Bat Herpesvirus Sequences

The BO herpesvirus putative DPOL coding sequences (CDS) and corresponding deduced amino acid (aa) sequences were determined using the ORF finder (https://www.ncbi.nlm.nih.gov/orffinder/, accessed on 2 October 2024) and validated by the BLASTX and BLASTP program (Basic Local Alignment Search Tool, www.ncbi.nlm.nih.gov/blast, accessed on 4 October 2024), respectively. The homology search for related herpesvirus sequences was performed using the standard BLASTN/BLASTX and BLASTP program. Pairwise nt and deduced aa sequence identities (%) were determined by BLASTN and BLASTP (using the ‘align two or more sequences option’), respectively, or the EMBOSS Needle (using sequence type option ‘DNA’ or ‘protein’, respectively, https://www.ebi.ac.uk/jdispatcher/psa/emboss_needle, accessed on 4 October 2024) program.

Phylogenetic analysis was performed by the maximum likelihood (ML) method using the MEGA11 software version 11.0.13 [34]. Briefly, multiple alignments of deduced aa sequences were carried out using the MUSCLE algorithm embedded in MEGA11, followed by construction of ML trees with the LG + G model of substitution (identified as the best-fit model of substitution by the ‘Find Best DNA/Protein Models (ML)’ tool in MEGA11) and 1000 bootstrap replicates. The clustering patterns of the BO herpesviruses were validated by ML analysis using other mathematical models of substitutions (JTT + G and WAG + G).

### 2.5. GenBank Accession Numbers

The BO herpesvirus DPOL sequences and the cyt-*b* sequences from herpesvirus-positive bats in St. Kitts were assigned GenBank accession numbers PQ497505-PQ497519 and PQ497520-PQ497534, respectively.

## 3. Results

**Figure 1:**
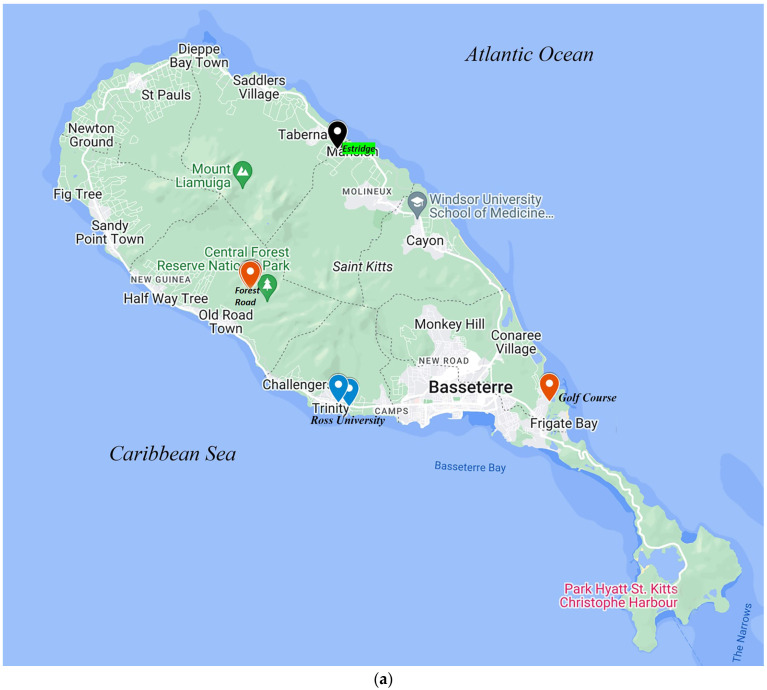

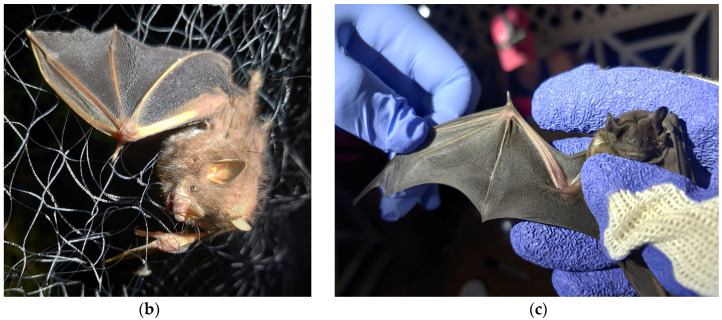
Map of the Caribbean Island of St. Kitts showing the capture locations of BO group-I and -II herpesvirus-positive bats (shown with red and blue pins, respectively). Bat BO27 (capture site shown with a black pin) yielded a low-quality (Phred value < 40) putative DNA polymerase sequence and was excluded from further analysis. The map of St. Kitts was adapted from https://www.google.com/maps (accessed on 2 October 2024) (**a**). In the present study, *Ardops nichollsi* (**b**) and *Molossus molossus* (**c**) tested positive for herpesviruses. The photographs were taken during sampling by Dr. Sarah Hooper, RUSVM (**b**,**c**).

In the present study, 16 (24.24%) of the 66 oral samples from apparently healthy bats on St. Kitts tested positive for herpesviruses by the *pan*-herpesvirus DPOL nested PCR assay [33]. The screening results were confirmed by BLASTN/BLASTX analysis of the partial DPOL sequences (~200 nt) obtained from the nested PCR products. Among the four bat species sampled in the study, herpesvirus DNA was detected in nine (75%, n = 12) and seven (28%, n = 25) samples from *A. nichollsi* and *M. molossus*, respectively, whilst all oral swabs obtained from *A. jamaicensis* (n = 17) and *B. cavernum* (n = 12) tested negative for herpesviruses. The nine *A. nichollsi* that tested positive for herpesviruses were captured in the rainforest (in all three mist nets placed along the forest road) during a single night (Table 1 and Figure 1). On the other hand, five of the seven herpesvirus-positive *M. molossus* were captured on or near the RUSVM campus, whilst one bat was sampled in Frigate Bay and the other at a private residence in Estridge estate (Table 1 and Figure 1).

Based on sequence identities and phylogenetic analysis of the partial DPOL CDS (~200 nt), 15 of the 16 BO herpesviruses were classified into two distinct groups, designated as BO-I (consisting of herpesviruses from 9 *A. nichollsi* and a single *M. molossus*) and -II (herpesviruses from 5 *M. molossus*) (Table 1 and Supplementary Figure S1). The BO27 DPOL sequence lacked quality (Phred value < 40) and was excluded from further analysis. The BO-I and -II herpesviruses shared nt sequence identities of 100% and 99.48–100%, respectively, within a group, and identities of ~57% between the groups. With other herpesviruses, the BO-I sequences shared maximum nt identities of 64.9% with a cognate DPOL sequence of *Miniopterus schreibersii* betaherpesvirus 2 (GenBank accession number KR608283) [16], followed by identities of ~63% with *Miniopterus fuliginosus* betaherpesvirus BatBHV-2 (AB517983) [35] and *Miniopterus schreibersii* betaherpesvirus 1 (JQ805139) [36]. On the other hand, the BO-II DPOL sequences shared maximum nt identities of 98.91–99.46% with a cognate sequence of betaherpesvirus MmolBHV1 (MN850443) from a *M. molossus* in the Lesser Antillean Island of Martinique [13].

To better understand the genetic diversity of bat herpesviruses in St. Kitts, we obtained longer DPOL CDS (~680 nt, encoding ~225 aa putative DPOL, corresponding to aa 641-aa 869 of DPOL of *Miniopterus schreibersii* betaherpesvirus 1) from 14 of the 16 BO strains. Samples BO27 and -35 lacked sufficient volumes and were not included in the analysis. The partial DPOL sequences (~225 aa) from BO-I and -II herpesviruses shared deduced aa identities of 99.5–100% and 99.56–100%, respectively, within a group, whilst identities of 65.8% were observed between the groups. By BLASTP analysis, the BO-I putative DPOL sequences shared maximum deduced aa identities of 67.71–69.10% with a cognate sequence of *Miniopterus schreibersii* betaherpesvirus 1, followed by identities of 66.37–67.81% with BatBHV-2 and <63% identities with other herpesviruses. The DPOL sequences from BO-II herpesviruses shared deduced aa identities of 65.37–66.09% and 63.91–64.66% with cognate sequence of *Miniopterus schreibersii* betaherpesvirus 1 and BatBHV-2, respectively, although maximum identities of 99.32–100% were observed with the partial DPOL sequence (146 aa, corresponding to aa 11-aa 156 of BO-II partial DPOL CDS) of MmolBHV1.

**Figure 2:**
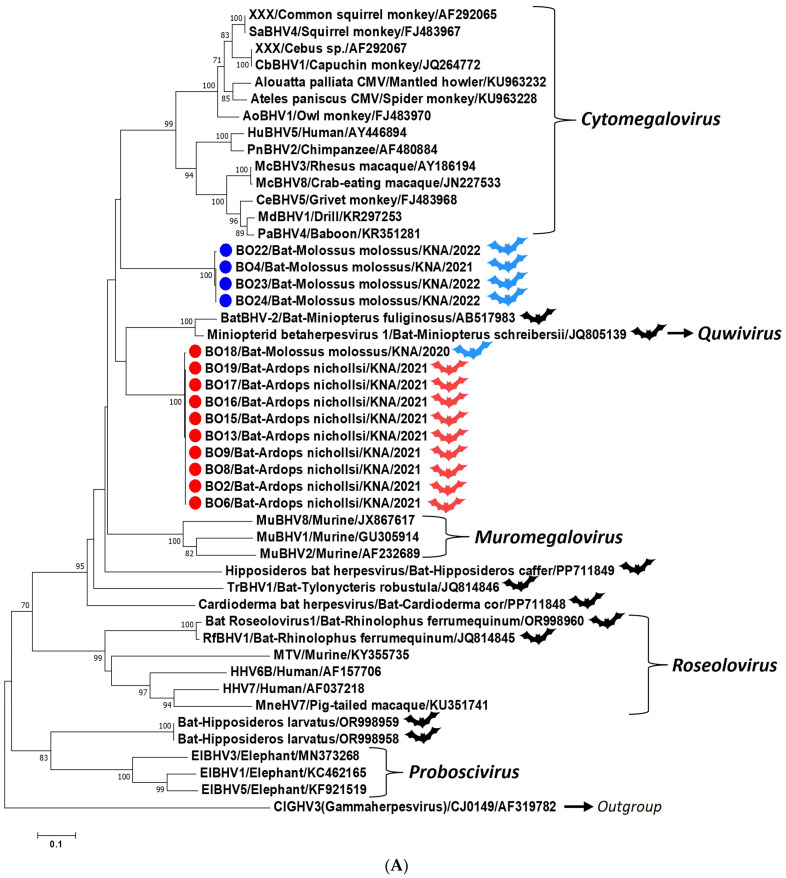

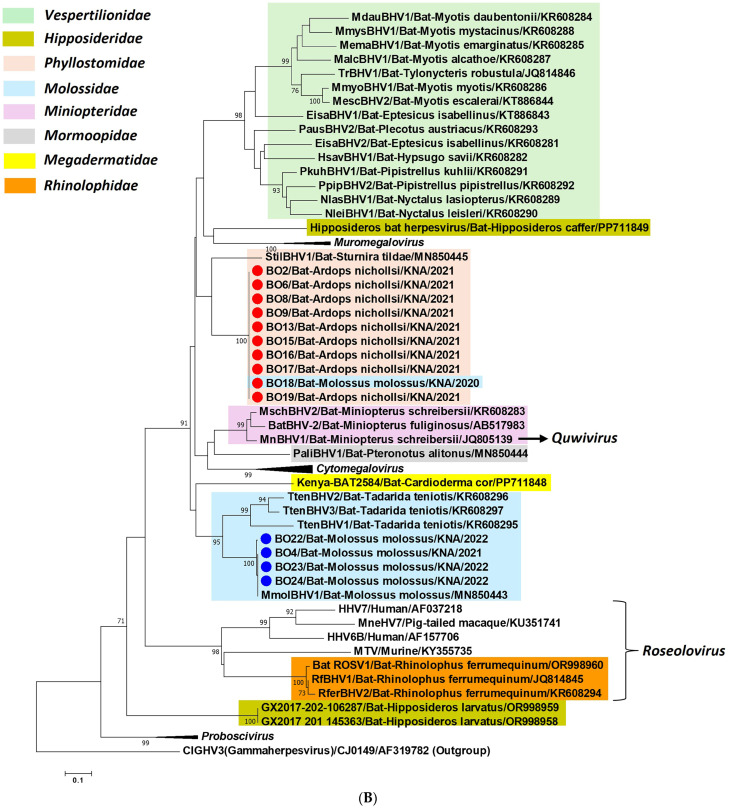
(**A**,**B**). Phylogenetic analysis of the partial deduced amino acid (aa) sequences (~225 aa, and ~150 aa putative DPOL sequences were analyzed in figure (**A**) and (**B**), respectively) of putative DNA polymerases of the BO-I and -II herpesviruses (shown with red and blue circles, respectively) from bats in St. Kitts with cognate sequences of other betaherpesviruses. (**A**): The *Ardops nichollsi*, *Molossus molossus*, and other chiropteran species are shown with red, blue, and black bat emojis, respectively. The bat emojis were obtained from https://creazilla.com/media/silhouette/7977573/bat (accessed on 10 October 2024) under a CC0 license. (**B**): The chiropteran families are mentioned to the upper left and highlighted with different colored boxes. (**A**,**B**): The recognized genera within the subfamily *Betaherpesvirinae* are shown with *italic* font. The ‘name of the virus/bat-species/country/year of sampling’ has been mentioned for the BO herpesviruses, whilst the name of the virus, the isolate, or the strain/host species/GenBank accession number is shown for the other betaherpesviruses. A member of the subfamily *Gammaherpesvirinae* (ClGHV3/CJ0149/AF319782) was used as the outgroup sequence. Bootstrap values < 70% are not shown. Scale bar, 0.1 substitutions per aa residue.

Based on phylogenetic analysis of the partial DPOL sequences (~225 aa), the BO-I and -II herpesviruses formed two distinct clusters within the subfamily *Betaherpesvirinae*, with *Miniopterus schreibersii* betaherpesvirus 1 (genus *Quwivirus*) and members of the genus *Cytomegalovirus*, respectively, as the nearest neighbor (Figure 2A). To date, complete/significant regions of DPOL CDS have been determined for a handful of bat betaherpesviruses, whilst partial DPOL sequences (~200 nt, or ~450 nt) are available for several betaherpesviruses from different chiropteran species [1,4,18]. Therefore, to rule out biases, we trimmed the BO DPOL sequences (~450 nt, encoding ~150 aa putative DPOL) and subjected them to phylogenetic analysis with a larger set of cognate sequences from bat betaherpesviruses (Figure 2B). Phylogenetically, most of the bat betaherpesviruses retained the host-specific clustering patterns (grouping within respective chiropteran families) (Figure 2B). The BO-I herpesviruses from *A. nichollsi* formed a distinct cluster near StilBHV1 from *Sturnira tildae* within the chiropteran family *Phyllostomidae*, whilst the BO-II herpesviruses and MmolBHV1 from *M. molossus* grouped together near the cluster of betaherpesviruses from *Tadarida teniotis* within the family *Molossidae* (Figure 2B). Interestingly, the BO-I cluster consisted of a single herpesvirus sequence from *M. molossus* within the *A. nichollsi* betaherpesvirus group (Figure 2B).

## 4. Discussion

To date, at least eight chiropteran species (*A. jamaicensis*, *A. nichollsi*, *B. cavernarum*, *Chiroderma improvisum*, *M. molossus*, *Monophyllus plethodon*, *Noctilio leporinus*, and *Tadarida brasiliensis*) have been reported from St. Kitts (~69 square miles, located in the Caribbean Lesser Antilles), of which *A. jamaicensis*, *A. nichollsi*, *M. molossus,* and *B. cavernarum* are more commonly sighted on the island [37]. In the present study, we reported high detection rates (24.24%, 16/66) of herpesviruses in bats on St. Kitts, corroborating previous observations on high prevalence of herpesviruses in bat populations from the other Caribbean Islands (Martinique and Puerto Rico) [13,28]. Among the four chiropteran species sampled in the study, herpesviruses were detected in *A. nichollsi* (75%, 9/12) and *M. molossus* (28%, 7/25). Based on analysis of partial DPOL sequences, we identified two genetically distinct herpesvirus groups (BO-I and -II) that were unique to *A. nichollsi* (except for a single *M. molossus*) and *M. molossus*, respectively, revealing significant genetic diversity among bat herpesviruses in a small, isolated geographical region (Figure 2A,B; Supplementary Figure S1). All the BO-I positive *A. nichollsi* were captured in the rainforest (using mist nets erected ~200 m apart along the forest road) during a single night, whilst the BO-II positive *M. molossus* were sampled on different dates from the same area (Table 1 and Figure 1). These observations indicated that the BO-I positive *A. nichollsi* might represent a single bat colony in the rainforest, whilst the BO-II bats might belong to the same *M. molossus* colony based around the RUSVM campus. To our knowledge, this is the first report on detection of herpesviruses from *A. nichollsi* (Antillean tree bats), expanding the host range of herpesviruses within the order Chiroptera.

Based on sequence identities and phylogenetic analysis, the partial DPOL sequences from the BO herpesviruses were more related to cognate sequences of betaherpesviruses than those of other viruses (Figure 2A,B). However, within the subfamily *Betaherpesvirinae*, the BO-I herpesviruses shared low deduced aa sequence identities (<70%) and phylogenetically formed a separate cluster, distinct from other betaherpesviruses, including chiropteran betaherpesviruses (Figure 2A,B). These findings suggest that the BO-I herpesviruses might represent a putative novel betaherpesvirus, although analysis of herpesvirus complete genomes would be required to confirm the observation. On the other hand, the BO-II DPOL sequences from *M. molossus* in St. Kitts were closely related (deduced aa identities of 99.32–100%) to the cognate sequence of putative novel betaherpesvirus MmolBHV1 from a *M. molossus* in the Lesser Antillean Island of Martinique (Figure 2B and Supplementary Figure S2). Phylogenetically, the BO-I (except strain BO18) and -II herpesviruses from St. Kitts exhibited species-specific (*A. nichollsi* and *M. molossus*, respectively) as well as family-specific (*Phyllostomidae* and *Molossidae*, respectively) clustering patterns (Figure 2B). Taken together, these observations indicated that putative novel betaherpesviruses BO-I and -II (and MmolBHV1) might be unique to chiropteran species *A. nichollsi* and *M. molossus*, respectively, corroborating the widely accepted hypothesis on host specificity of betaherpesviruses [1,6,10,12,13,14,15,16,17] and revealing the high genetic diversity of betaherpesviruses between bat species belonging to different chiropteran families.

The close genetic relationship between the BO-II herpesviruses and MmolBHV1 indicated possible transmission of herpesviruses by bat movement between the Caribbean Islands, which might not be unusual, as (i) *M. molossus* has been shown to fly long distances. There is evidence that oceanic straits do not pose a significant barrier to the movement of these bats in the Lesser Antilles [38], and (ii) St. Kitts and Martinique are separated by a distance of ~350 km, with the islands of Dominica, Guadeloupe, and Montserrat located in between (Supplementary Figure S2). Therefore, surveillance for widely prevalent viruses, such as herpesviruses, might be useful in tracking bat migration routes, with possible implications on better understanding of bat ecology, and forecasting potential spread of highly pathogenic bat-borne viruses [11,12].

Although herpesviruses are believed to be host-specific and appear to coevolve with their host, there is evidence for herpesvirus cross-species transmission events (including zoonoses, anthroponoses, and even infection of distantly related host species), sometimes resulting in fatal disease in non-definitive hosts, with possible implications on human and animal health and on wildlife conservation [5,6,7]. Between the orthoherpesvirus subfamilies, host switching events appear to be less prominent among the betaherpesviruses, which, however, might be due to undersampling or undiscovered viruses [1,5,6,7]. To date, only a few studies have provided evidence on interspecies transmission of betaherpesviruses within the order *Chiroptera* [14,17,20]. In the present study, a single *M. molossus* betaherpesvirus (BO18) clustered with the BO-I *A. nichollsi* betaherpesviruses (Figure 2A,B), indicating possible cross-species transmission of herpesviruses between the chiropteran families *Phyllostomidae* and *Molossidae*. On the other hand, it might be possible that the BO-I herpesviruses are circulating in both *A. nichollsi* and *M. molossus* on St. Kitts and were underrepresented in *M. molossus* in the present study (due to a small sample size), although typically, betaherpesviruses have been shown to be specific to chiropteran families [10,12,13,14,15,16,17].

The evidence for cross-species transmission of betaherpesviruses between *A. nichollsi* and *M. molossus* was interesting, as the two bat species have different life histories and ecology [25,37,39]. The *A. nichollsi* (frugivorous bats) have only been observed roosting in pairs or small groups in the branches/foliage of trees in forests and frequently move between roosts, whereas *M. molossus* (insectivorous bats) primarily roost in man-made structures, although they can roost in hollow trees and palm trees in non-cluttered environments [25,37,39]. Additionally, since *M. molossus* are high-flying, aerial bats, they have difficulty maneuvering in rainforests (where the *A. nichollsi* were captured in the present study) [25,37,39,40]. Therefore, it is plausible that the cross-species transmission event between *A. nichollsi* and *M. molossus* might have occurred through a common freshwater source, or a contaminated fomite near the water body. Certain herpesviruses have been shown to remain stable and retain infectivity in freshwater for up to three weeks, and herpesvirus cross-species transmission events have been reported in captive animal species with shared water sources [41,42]. The different bat species captured during the present study were likely to access the same water bodies, as there are limited sources of freshwater available to wildlife on St. Kitts, and only a few of the water bodies meet the requirements for bats to be able to utilize them (unobstructed water bodies that are pooled or slow-moving and of conducive lengths).

Contamination of samples was unlikely, as (i) *M. molossus* BO18 and the *A. nichollsi* were sampled at different locations (Frigate Bay and Forest Road on 21 December 2020 and 8 January 2021, respectively) (Table 1 and Figure 1), (ii) the sampling equipment was thoroughly disinfected between sites, (iii) the samples were processed in the laboratory on different dates, and (iv) the bat species nature was confirmed by analysis of cyt-*b* sequences from respective samples using two different sets of primers [24,32].

Taken together, the present study (i) reported herpesviruses for the first time in Antillean tree bats (*A. nichollsi*), adding a new host species within the order *Chiroptera*, (ii) identified putative novel betaherpesviruses that might be unique to bat species (*A. nichollsi* and *M. molossus*), indicating virus–host coevolution, (iii) provided evidence for possible interspecies transmission of betaherpesviruses between chiropteran families, contradicting the established hypothesis on host specificity of betaherpesviruses, and (iv) indicated possible transmission of herpesviruses by bat movement between the Caribbean Islands. Although these observations provided important insights on bat herpesviruses in an isolated geographical region, the present study was limited to a small island (St. Kitts), warranting large-scale studies on molecular detection and genetic diversity/evolution of herpesviruses in various chiropteran species across the Caribbean region.

The present study also had other limitations: (i) the study was based on a small sample size, which might not represent the overall prevalence and genetic diversity of bat herpesviruses circulating in various chiropteran species on St. Kitts, (ii) bat samples were not available in sufficient volumes for isolation of herpesviruses or determination of herpesvirus whole genome sequences by next generation sequencing technologies, and (iii) we failed to amplify the complete/nearly complete DPOL and other relevant herpesvirus genes, such as gB, from the bat samples using different sets of degenerate primers that had been published [13,15,16,43] or designed in this study from reference herpesvirus sequences, including those from bats.

## Figures and Tables

**Table 1:** Details of the bats that tested positive for herpesviruses on St. Kitts Island.

Bat/Sample	*Chiropteran Species* ^1^	Sampling Date	Sampling Location in St. Kitts	Herpesvirus Group ^2^
BO2	*Ardops nichollsi*	8 Jan 2021	Forest road (Mist net A) ^3^	BO group-I
BO4	*Molossus molossus*	7 Jan 2021	RUSVM campus	BO group-II
BO6	*Ardops nichollsi*	8 Jan 2021	Forest road (Mist net B) ^3^	BO group-I
BO8	*Ardops nichollsi*	8 Jan 2021	Forest road (Mist net C) ^3^	BO group-I
BO9	*Ardops nichollsi*	8 Jan 2021	Forest road (Mist net C) ^3^	BO group-I
BO13	*Ardops nichollsi*	8 Jan 2021	Forest road (Mist net A) ^3^	BO group-I
BO15	*Ardops nichollsi*	8 Jan 2021	Forest road (Mist net C) ^3^	BO group-I
BO16	*Ardops nichollsi*	8 Jan 2021	Forest road (Mist net C) ^3^	BO group-I
BO17	*Ardops nichollsi*	8 Jan 2021	Forest road (Mist net A) ^3^	BO group-I
BO18	*Molossus molossus*	21 Dec 2020	Private residence near golf course in Frigate Bay	BO group-I
BO19	*Ardops nichollsi*	8 Jan 2021	Forest road (Mist net C) ^3^	BO group-I
BO22	*Molossus molossus*	27 May 2022	RUSVM campus	BO group-II
BO23	*Molossus molossus*	27 May 2022	RUSVM campus	BO group-II
BO24	*Molossus molossus*	27 May 2022	RUSVM campus	BO group-II
BO27	*Molossus molossus*	1 Oct 2022	Private residence, Estridge estate	Not determined ^4^
BO35	*Molossus molossus*	1 Apr 2022	Private pool, ~2 km north of RUSVM campus	BO group-II

^1^ Based on morphometric identification by an experienced field biologist (S.H.) and BLASTN analysis of mitochondrial DNA cytochrome-*b* sequences. ^2^ Based on sequence identities and phylogenetic analysis of partial DPOL sequences (~200 nt), the bat herpesviruses from St. Kitts (BO strains) were classified into two distinct groups, designated as BO group-I and -II. ^3^ The bats were captured in the Central Reserve Forest (in three mist nets erected along the forest road) during the night of 8 January 2021. ^4^ By BLASTN analysis, the partial DPOL sequence (~200 bp) of BO27 shared maximum homology with chiropteran betaherpesviruses. However, the BO27 sequence lacked quality (Phred value < 40) and was excluded from further analysis. Abbreviations: *nt*, nucleotide; *RUSVM*, Ross University School of Veterinary Medicine.

## Data Availability

The data presented in this study are available in this article and Supplementary Figures S1 and S2.

## Supplementary Materials

The following supporting information can be downloaded at https://www.mdpi.com/article/10.3390/microorganisms12122603/s1. Figure S1: Phylogenetic analysis of the partial DPOL CDS (~200 nt) of the bat herpesviruses from St. Kitts; Figure S2: Map of the Caribbean Lesser Antilles showing the geographical locations of St. Kitts and Martinique (shown with a red and a dark teal arrow, respectively).
