# Supplementary material for: Novel Betaherpesviruses in Neotropical Bats on the Caribbean Island of St. Kitts: First Report from Antillean Tree Bats (Ardops nichollsi) and Evidence for Cross-Species Transmission
Source: Microorganisms. 2024 Dec 16;12(12):2603. doi: 10.3390/microorganisms12122603 (PMC11677976; doi:10.3390/microorganisms12122603)
Supplement: Supplementary file 1 [file microorganisms-12-02603-s001.zip › Supplementary files/Supplementary figure S1.pptx]

## Slide 1
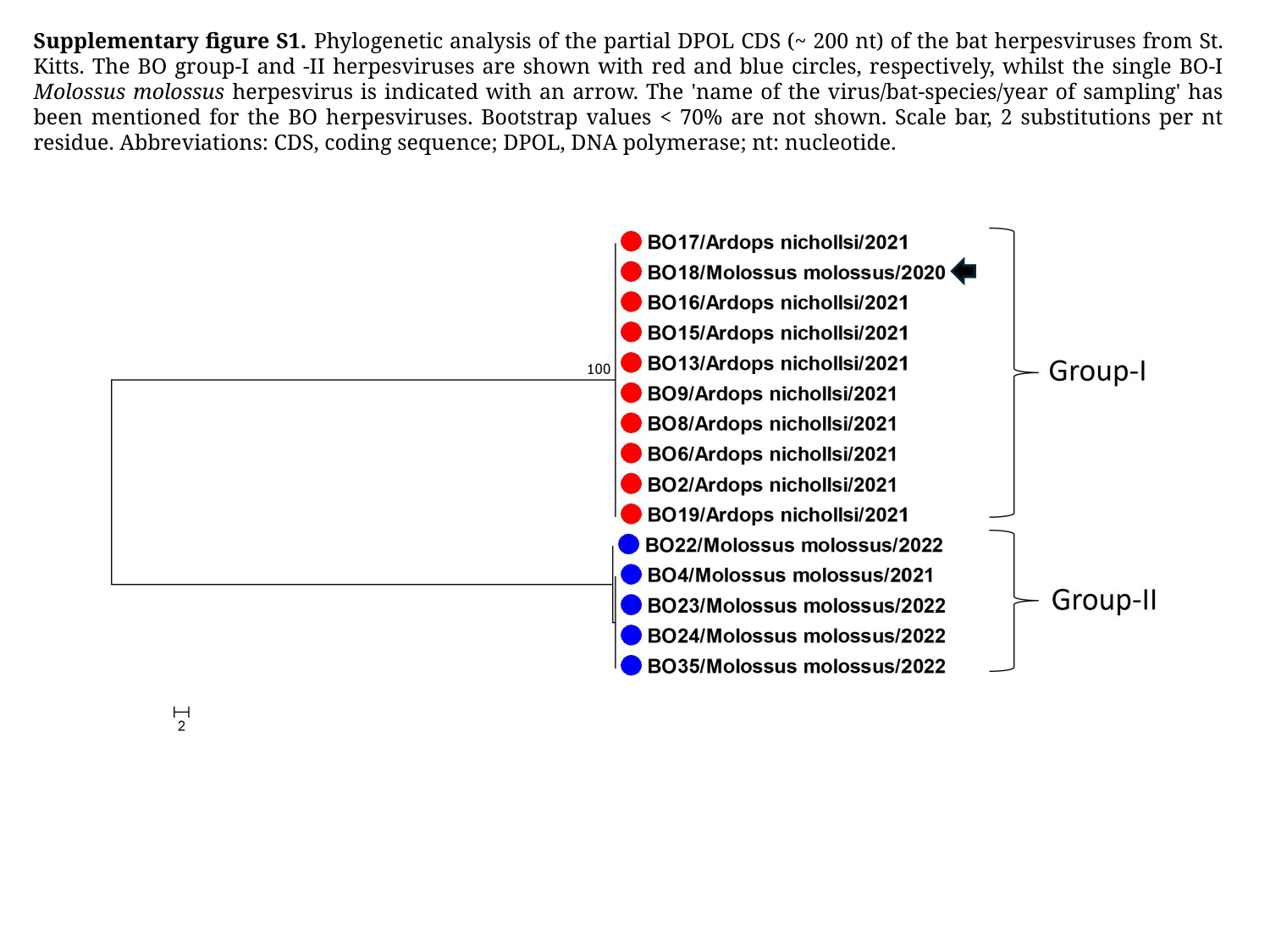

Supplementary figure S1. Phylogenetic analysis of the partial DPOL CDS (~ 200 nt) of the bat herpesviruses from St. Kitts. The BO group-I and -II herpesviruses are shown with red and blue circles, respectively, whilst the single BO-I Molossus molossus herpesvirus is indicated with an arrow. The 'name of the virus/bat-species/year of sampling' has been mentioned for the BO herpesviruses. Bootstrap values < 70% are not shown. Scale bar, 2 substitutions per nt residue. Abbreviations: CDS, coding sequence; DPOL, DNA polymerase; nt: nucleotide.
