# Supplementary material for: Novel Betaherpesviruses in Neotropical Bats on the Caribbean Island of St. Kitts: First Report from Antillean Tree Bats (Ardops nichollsi) and Evidence for Cross-Species Transmission
Source: Microorganisms. 2024 Dec 16;12(12):2603. doi: 10.3390/microorganisms12122603 (PMC11677976; doi:10.3390/microorganisms12122603)
Supplement: Supplementary file 1 [file microorganisms-12-02603-s001.zip › Supplementary files/Supplementary figure S2.pptx]

## Slide 1
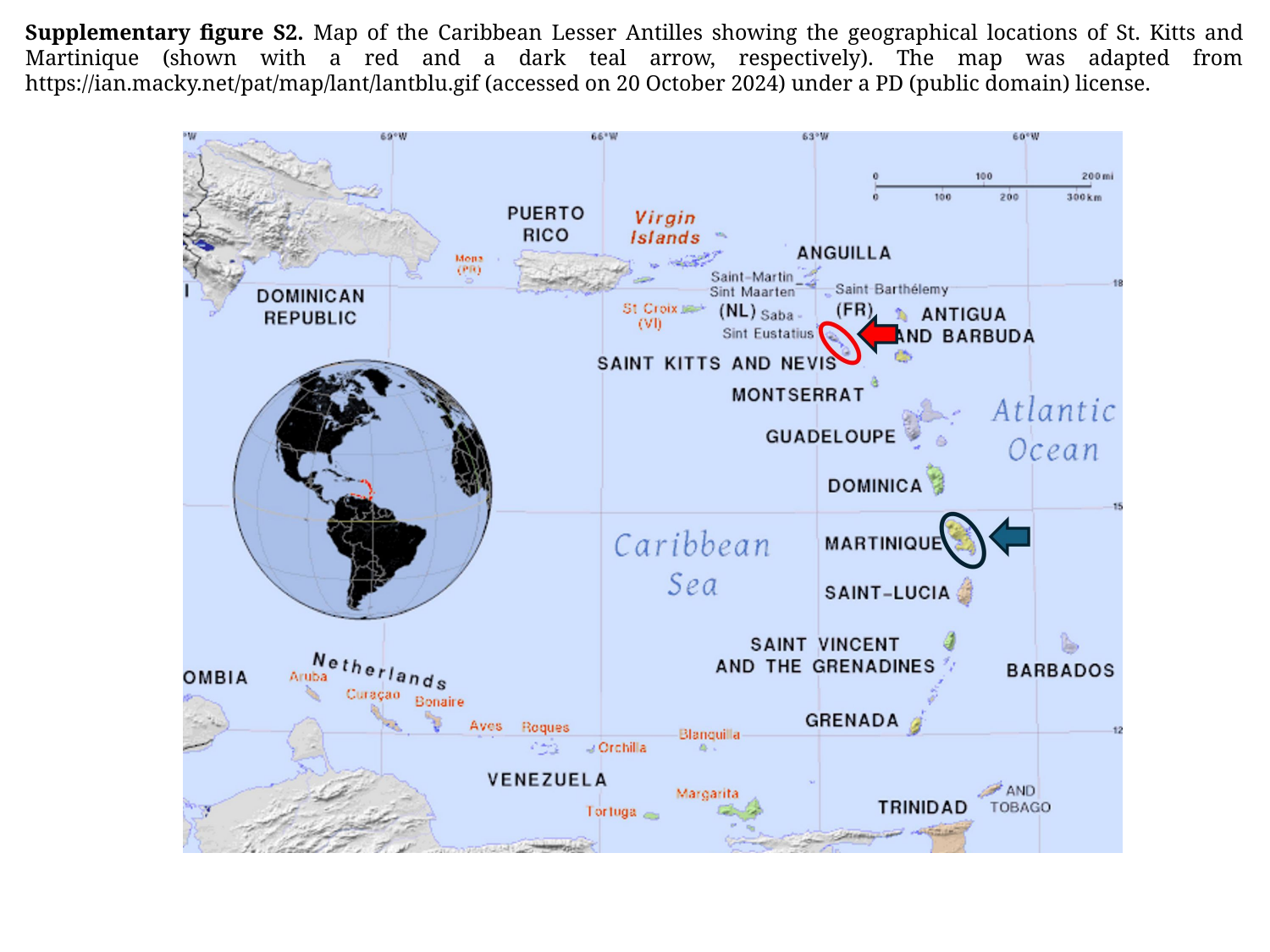

Supplementary figure S2. Map of the Caribbean Lesser Antilles showing the geographical locations of St. Kitts and Martinique (shown with a red and a dark teal arrow, respectively). The map was adapted from https://ian.macky.net/pat/map/lant/lantblu.gif (accessed on 20 October 2024) under a PD (public domain) license.
